# Feasibility and Preliminary Efficacy of the Eating Advice to Students (EATS) Brief Web-Based Nutrition Intervention for Young Adult University Students: A Pilot Randomized Controlled Trial

**DOI:** 10.3390/nu11040905

**Published:** 2019-04-23

**Authors:** Megan C Whatnall, Amanda J Patterson, Simon Chiu, Christopher Oldmeadow, Melinda J Hutchesson

**Affiliations:** 1 School of Health Sciences, Faculty of Health and Medicine, and Priority Research Centre for Physical Activity and Nutrition, University of Newcastle, Callaghan 2308, Australia; megan.whatnall@uon.edu.au (M.C.W.), amanda.patterson@newcastle.edu.au (A.J.P.); 2 Clinical Research Design and Statistics Support Unit, Hunter Medical Research Institute, New Lambton Heights 2305, Australia; simon.chiu@hmri.org.au (S.C.), christopher.oldmeadow@hmri.org.au (C.O.)

**Keywords:** young adults, university students, college students, nutrition, eating behavior, brief, web-based intervention, eHealth

## Abstract

Young adult university students are a priority population for nutrition intervention. This study assessed the feasibility and preliminary efficacy of the EATS (Eating Advice to Students) brief (i.e., single use) web-based nutrition intervention for young adult university students. A 3-month pilot randomized controlled trial (RCT) was conducted with 124 students aged 17–35 from the University of Newcastle, Australia. Participants were randomized to EATS (*n* = 62) or attention control (*n* = 62). EATS aimed to improve four target eating behaviors (vegetables, fruit, discretionary foods, breakfast). Primary outcomes were feasibility (recruitment, retention, usage, program acceptability). Recruitment and retention numbers were recorded, the program acceptability was assessed by a process evaluation survey and the website usage was objectively tracked. Preliminary efficacy was assessed via changes in diet quality (primary), fruit, vegetables, discretionary foods and breakfast intake, measured using Food Frequency Questionnaire. Recruitment was completed in five weeks. Retention was 73% (90/124) at 3-months. Intervention participants used EATS 1.5 ± 1.0 times. Satisfaction with EATS was rated at 4.04 ± 0.74 (maximum five). Intervention participants significantly decreased the percentage energy/day from discretionary foods compared with control (−4.8%, 95%CI −8.6, −1.1, *p* = 0.012, *d* = −0.34). No significant between-group differences were observed for diet quality, fruit, vegetable or breakfast intakes. EATS demonstrated high feasibility, particularly for reach and acceptability. The university setting and a brief web-based intervention show promise in engaging young adults to improve their eating behaviors.

## 1. Introduction

Young adult (17–35 years) university students are in a transitional life stage and setting, where major life changes can have a negative impact on lifestyle behaviors. Changes in living situation, employment, social life, and the development of self-identity and self-efficacy are some of the impacting factors, on top of university study [1,2]. Unhealthy eating behaviors are of particular concern among young adult university students due to their high prevalence [3] and likelihood of tracking into middle age [4], and because of the associations with poorer mental health [5,6], lower academic achievement [7] and increased risk of weight gain and other chronic disease risk factors [8,9]. Therefore, there is a clear need for nutrition intervention in the university setting. 

Developing effective nutrition interventions for university students is challenging as they are a unique population group and difficult to engage [10,11]. Further, the heterogeneity of existing nutrition interventions in this group makes determining the most effective intervention difficult [3,11]. A systematic review exploring nutrition, physical activity and weight management interventions among university students found that the more effective nutrition interventions targeted nutrition only rather than multiple behaviors, were no longer than a university semester (approximately 12 weeks) in duration, and provided feedback on behavior rather than simply education [11]. Utilizing eHealth may also be effective in this group, with a growing number of studies finding that eHealth interventions (e.g., web-based and text message interventions) have significantly improved eating behaviors among university students, predominantly fruit and vegetable intakes [12,13,14]. The accessibility of eHealth and flexibility in terms of time commitment are likely contributors to their effectiveness and appeal within young adult university students [12,15]. There is also the capability with eHealth technology to differentiate interventions, i.e., personalize. This comes back to the need for interventions which are targeted to the population group and tailored to individual characteristics and determinants of behavior, including barriers and enablers [2].

A major barrier to healthy eating and participation in behavior change interventions for young adult university students is lack of time [2]. This is because they are often juggling a combination of work, study, sport, social and family commitments, and because healthy eating is typically given lower priority than these other commitments [2]. As such, brief interventions, i.e., those involving minimum input and contact to achieve significant and sustainable effect, could be an effective intervention approach in this group [16,17]. Brief web-based interventions have shown efficacy to significantly reduce alcohol intake among university students [18] and young adults [15]. In addition, a systematic review of brief (defined as single session) nutrition interventions in adults found that, when they are tailored and instructional, brief interventions can significantly improve eating behaviors in the short term, including increasing fruit and vegetable intake, reducing fat intake, and improving overall diet quality [17].

Combining this evidence, and guided by a participatory research model [19], the Eating Advice To Students (EATS) brief web-based nutrition intervention for young adult university students was developed [20]. In this study brief intervention refers to a single use intervention. The aims of this randomized controlled trial (RCT) were to:

1. Evaluate the feasibility of a brief web-based nutrition intervention for young adult (17–35 years) university students, including program acceptability and demand (usability and appropriateness), usage, recruitment and retention. 

2. Estimate the impact of a brief web-based nutrition intervention for young adult (17–35 years) university students on eating behaviors after three months compared with an attention control group. 

## 2. Materials and Methods 

### 2.1. Study Design

A pilot RCT was conducted with participants randomly allocated to the EATS intervention or attention control group. Program acceptability measures were collected post intervention completion, and intervention usage measures were collected in real time. Impact evaluation measures were collected at baseline and three months after baseline. The trial was registered with the Australian New Zealand Clinical Trials Registry, Number ACTRN12618000118202p. Full details of the intervention development and the trial methods have been previously published [20]. Ethics approval was obtained from the University of Newcastle Human Research Ethics Committee (H-2017-0404). All participants gave informed consent prior to participation. The design, conduct and reporting adhere to the Consolidated Standards of Reporting Trials (CONSORT) guidelines for reporting parallel group randomized trials [21].

### 2.2. Participants and Recruitment

Inclusion criteria were: Aged 17–35 years and currently enrolled as a student at the University of Newcastle, Australia. Exclusion criteria included having a medical condition requiring a prescribed diet (e.g., diabetes) and studying outside of Australia (i.e., Singapore campus students and external/distance students located outside of Australia). Study recruitment was from 20 February to 29 March 2018, spanning 38 days or approximately five weeks. Recruitment was via posts on the university social media pages (Facebook and Twitter), and posters and digital signage displayed across university campuses. Recruitment was also supported by the project steering committee and the University Health Promotion Working Group, who were asked to promote the study via personal networks within the university using the recruitment materials provided by the researchers. These recruitment methods were used throughout the recruitment period, however due to insufficient numbers a secondary method of recruitment was also used and the recruitment period was extended by 11 days (19 to 29 March). Recruitment was stopped at 29 March so that the trial would not extend beyond the end of the semester. University teaching staff were emailed and asked to promote the study in class or via the online learning management system using the recruitment materials provided by the researchers. Follow up was from 15 May to 15 July 2018. Participants received $AU10 gift vouchers as a reimbursement for their time; one after completing baseline measures and a second after completing follow-up measures. All measures were collected via online surveys hosted in Qualtrics (www.qualtrics.com). 

### 2.3. Intervention: Eating Advice to Students

EATS is a web-based intervention which aims to assist young adult university students to improve their diet quality, including specifically targeting consumption of fruits, vegetables, discretionary foods (i.e., energy-dense nutrient poor foods) [22], and breakfast. EATS was designed as a brief or single-use intervention. The four components of EATS include (1) a brief screening quiz providing personalized feedback on eating behaviors and barriers to healthy eating; (2) provision of information, tips, and strategies for each target behavior and two guided exercises to facilitate behavior change; (3) goal setting; and (4) creating strategies. The website also provides resources including links and downloads to other reputable sources for further information, for example a cookbook with cheap and healthy recipe ideas. The development of EATS was based on the PRECEDE-PROCEED participatory research model [19], and draws on social cognitive theory (SCT) [23] and the theory of planned behavior (TPB) [24]. Intervention participants were sent personal login details for EATS via email at the time of randomization, with instructions to access on a single occasion within the next week.

### 2.4. Attention Control Group 

Control group participants completed Thrive, an existing brief web-based alcohol intervention designed for university students [25], and received access to the EATS intervention after completing follow-up measures. Control participants were sent details for accessing Thrive via email at the time of randomization, with instructions to access on a single occasion within the next week. 

### 2.5. Measures

#### 2.5.1. Process Evaluation Measures

The primary outcomes were the process evaluation measures; recruitment and retention, intervention usage and program acceptability. 

Recruitment was assessed by recording the number of individuals interested in participating in relation to the recruitment strategies used, and by asking participants how they heard about the study during the baseline survey. Retention was assessed as the number of participants completing the process evaluation and follow-up measures. The number of reminders used during data collection was also recorded. 

Intervention usage was objectively tracked using Google Analytics and the Qualtrics Survey Platform. This included the duration of website use, number of pages viewed and links accessed, whether participants accessed the quiz, goal setting, and creating strategies components, and the number of goals and strategies set. 

Program acceptability was evaluated using a survey developed for the study, including satisfaction, usability, and appropriateness. Participants were asked to rate the EATS components (quiz, goal setting and creating strategies) and the website overall on a 5-point Likert scale from strongly agree (= 5) to strongly disagree (= 1) for usefulness, relevance, usability, and ability to motivate. Participants were also asked to rate their overall satisfaction with EATS, from very satisfied (= 5) to very unsatisfied (= 1), whether the intervention met their expectations, from strongly agree (= 5) to strongly disagree (= 1), and their opinion on the time taken to complete the intervention *(too much time, a good amount of time, too little time).* The participants were also asked open-ended questions about their likes and dislikes and suggestions for improvement for EATS, as well as reasons for not visiting webpages or using intervention components where relevant. 

#### 2.5.2. Impact Evaluation Measures 

Diet quality was a primary outcome of preliminary efficacy, where all other impact evaluation measures were secondary outcomes. 

Dietary intake was assessed using the Australian Eating Survey Food Frequency Questionnaire (AES FFQ), a self-administered 120-item semi-quantitative FFQ previously validated in a healthy adult population [26]. Nutrient intakes were calculated using the Australian AusNut 1999 (All Foods) Revision 17 food composition database, primarily and AusFoods (brands) Revision 5. Participants were asked to report their usual intake over the previous three months. Diet quality (Australian Recommended Food Score (ARFS)) was derived from FFQ responses. ARFS is calculated using 70 items from the AES FFQ relating to intake of fruit, vegetables, meat/flesh foods, non-meat/flesh protein foods, breads and cereals, dairy foods, water and spreads/sauces [27]. The ARFS is a summation of points for each item, with most items scoring one point for a consumption frequency of ≥once per week, and the total score range of zero to 73 points. A higher ARFS reflects higher diet quality; greater variety, more optimal nutrient intakes and closer alignment with the Australian Dietary Guidelines [22]. Intake of fruits, vegetables, discretionary foods, and breakfast were also determined from FFQ responses. These are reported as grams per day, percentage of daily energy intake and the ARFS subscale for fruits and vegetables, percentage of daily energy intake for discretionary foods, and days per week for breakfast *(Never, 1–2 days, 3–4 days, 5 or more days).*

Alcohol intake was assessed using two items from the New South Wales Adult Population Health Survey [28]; frequency of alcohol consumption and number of standard drinks usually consumed per drinking occasion. 

Self-efficacy for performing the target eating behaviors was assessed by asking participants to rate their confidence for each behavior on a scale of Not at all confident (= 1) to Very confident (= 4), using questions derived from the validated Project EAT II Survey for Young Adults [29]. 

Quality of life was assessed using the Quality of Life Enjoyment and Satisfaction Questionnaire short form, previously validated in a general adult population [30], and well-being was assessed using the World Health Organization-Five Well-being Index [31], previously validated as an outcome measure in clinical trials among adolescents and adults [32].

#### 2.5.3. Measures of Potential Contamination

The follow-up survey included three items that assessed what, if anything, participants had used to help them change their eating behavior to assess the potential contamination of intervention effects. Participants were asked whether they had made changes to their eating behavior during the three months since baseline (i.e., the study period) *(Yes or No)*. If yes, they were asked to describe the changes *(open-response question)*, and to indicate what they had used to help them change their behavior *(EATS, Other research study, Other program e.g., Lite n’Easy or Weight Watchers, Other sources of nutrition information e.g., website, smartphone app, Other, None of the above).*

### 2.6. Sample Size 

A sample size calculation was not performed, as this was a pilot RCT. A total sample size of 126 (i.e., 63/group) was set based on available time for recruitment, study funding, and to allow for greater diversity within the sample by exceeding the median sample size of 30.5 among previous pilot trials [33]. A lower limit of 30% male participants was set as males are often underrepresented in health behavior research [34]. To maximize the proportion of male participants, females were waitlisted once the proportion of females reached 70% of the target sample size. However, the proportion of 30% males was unable to be reached, and female participants were invited from the waitlist from 22 March (i.e., one week before the recruitment period ended on 29 March). 

### 2.7. Randomization

Allocation was stratified by gender (female; male and other gender identity). Allocation sequences within strata were generated by a statistical software program, using permuted block randomization with random block sizes of 2 and 4. Randomization was generated by a statistician external to the study. Randomization was generated and the allocation sequence was provided to the research team prior to study commencement. One member of the research team was responsible for allocating participants to groups based on the allocation sequence. Participants were allocated to groups after completing baseline measures. The research team were not blinded to group allocations or outcomes assessment, however all data were collected via self-report online surveys. 

### 2.8. Data Analysis Plan

Data were analyzed using Stata software version 14.1. Program acceptability measures are reported as means and standard deviations for quantitative questions, with open question responses described qualitatively. Intervention usage measures are reported as means and standard deviations. Attempts were made to match intervention usage data from the different sources to individual participants, so that this could be compared with the self-report data for intervention usage and program acceptability for consistency. The impact of the EATS intervention compared with the control was determined using an intention-to-treat approach (all participants who completed baseline measures and were randomized) and for completers only (participants who provided data at three months). A sensitivity analysis was also conducted including participants reporting plausible energy intake only, determined using the Goldberg cutoffs [35] as detailed in the Supplementary Material. The difference between the intervention and control groups for the change from baseline to three months was assessed for all outcomes using linear mixed models. The models included fixed effects for treatment and time, and their interaction, and random intercepts for participants. The interaction term assessed the difference in change from baseline to follow-up between intervention and control groups, and is presented together with 95% confidence intervals. 

## 3. Results

### 3.1. Recruitment 

**Figure 1:**
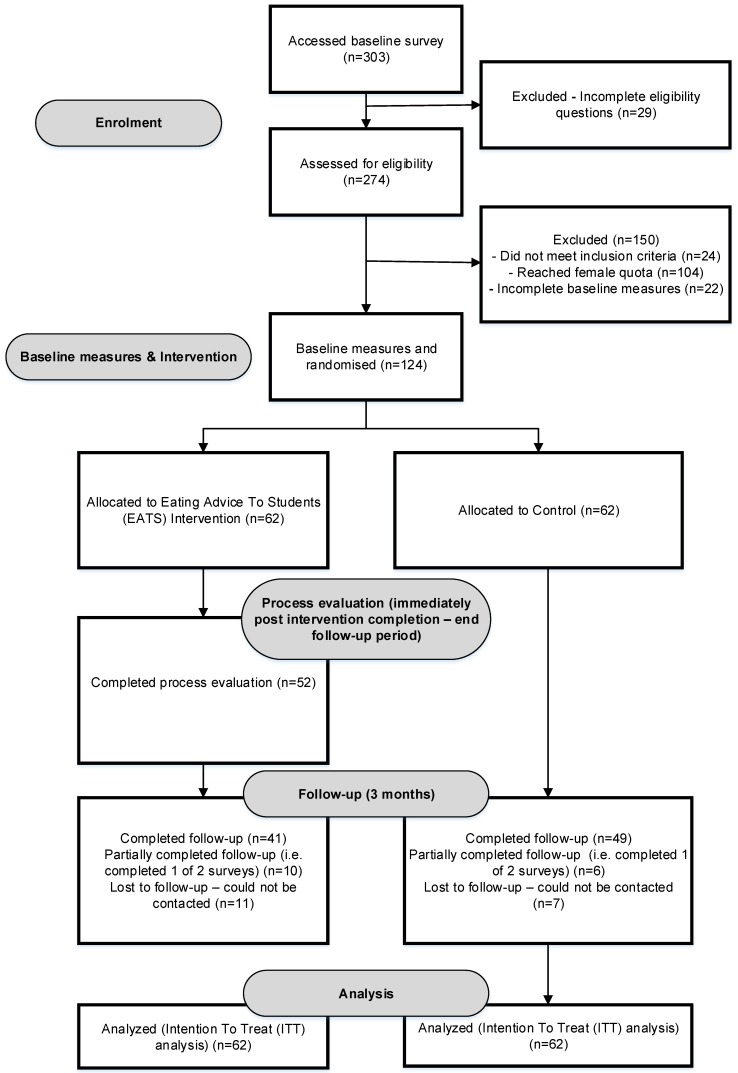
CONSORT flow chart describing participant progress through the Eating Advice to Students (EATS) pilot randomized controlled trial.

**Figure 2:**
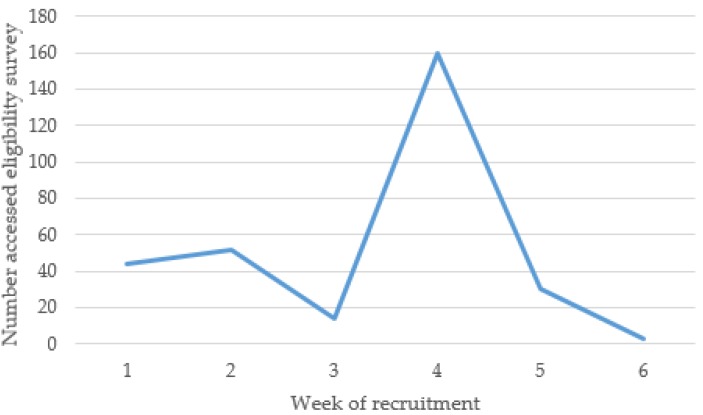
Number of individuals interested in participating in the EATS pilot randomized controlled trial according to recruitment week and recruitment strategies used.

A total of 303 individuals expressed interest in participating (Figure 1). A total of 274 individuals were assessed for eligibility, of which 124 were randomized into the EATS or control group. Among the 124 participants enrolled in the study, the most successful recruitment method was via the University social media pages (41%), followed by promotion via the university teaching staff (36%) and posters displayed across university campuses (17%). Less successful recruitment methods were hearing about the study from a friend (10%), and computer screen savers displayed across university campuses (2%). When comparing the number of interested individuals with the recruitment week and strategies used (Figure 2), the greatest number of individuals were interested (i.e., accessed the eligibility survey) in week four.

### 3.2. Participants at Baseline

Baseline data for the 124 randomized participants are summarized in Table 1. The mean age of participants was 22.4 ± 4.0 years and the percentage of male participants was 27.4%. Most participants were Australian born (83.1%), never married (85.5%) and living in rented accommodation (41.9%). The majority were undergraduate students (87.1%) and receiving financial support (76.6%). Participants mean Australian Recommended Food Score was 30.5 ± 9.8 (Max score = 73). The mean percentage energy/day from discretionary foods was 36.6%, while 33.1% of participants reported consuming breakfast less than five days/week. Participants mean well-being score on the WHO-5 Well-being Index was 14.7 ± 4.3 out of a maximum score of 25, and mean quality of life score on the Quality of Life Enjoyment and Satisfaction Questionnaire was 51.3 ± 7.5 out of 70. 

### 3.3. Retention 

Of the 124 participants enrolled in the study, 90 provided complete data at three months (73%). A further 16 participants provided partial data (i.e., completed one of the two follow up surveys; AES FFQ or other questions), resulting in data from 86% of participants at follow up. Of the 106 participants who provided all or partial data at three months, 48% completed both surveys without a reminder, while 13%, 8%, 4% and 4% completed both surveys following one, two, three and four reminders, respectively. The remaining 23% were reminded five or more times, including 8% who completed both surveys and 15% who completed one of the two surveys. Baseline differences between those who completed follow up and those who did not were assessed. Non-completers were more likely to be enrolled in enabling and non-award courses (*p* = 0.037), in their first year of study (*p* = 0.008), have higher BMI (*p* = 0.013), have a higher percentage energy/day from discretionary foods (*p* = 0.001), have a lower WHO-5 wellbeing score (*p* = 0.014), and have lower self-efficacy to reduce sugar sweetened drinks intake (*p* = 0.020). 

### 3.4. Intervention Usage 

A total of 58 of the 62 intervention participants (93.6%) logged-on to the EATS website. The mean number of website sessions was 1.5 ± 1.0 (Range 1–6), for a mean of 13.5 ± 15.6 min per session (Range 0.02–88.6 min). Most participants (69.0%) used EATS within a single session. The average number of days between being randomized and accessing EATS was 2.9 ± 4.3 days, with 86.2% accessing EATS within seven days. Of those who completed EATS over more than one session (31.0%), the average number of days between sessions was 2.8 ± 4.0 days. The average time spent using the website in total per participant was 16.8 ± 17.1 min. There were 10 webpages included in the website and three components to access (quiz, goal setting, and creating strategies). On average, participants viewed 6.6 ± 4.0 webpages and accessed 1.6 ± 1.0 components per session, with 8.6 ± 4.8 webpage views and 2.0 ± 1.0 components accessed in total per participant over all sessions. There were 7 resources that could be accessed via the website. On average participants accessed 0.4 ± 1.0 resources per session and 0.7 ± 0.9 resources in total over all sessions. 

The quiz was the most accessed component, with 50 participants (80.6%) accessing and completing the quiz. The goal-setting component was accessed by 35 participants (56.5%), of which 28 completed it. Participants set on average three goals (Range 0–7). The creating strategies component was accessed by 28 participants (45.2%), of which 19 completed it. Participants set on average two strategies (Range 0–9). The majority of participants accessed the website via a desktop or laptop computer (63.8%), followed by mobile (29.3%) and tablet (6.9%).

Sixty-one of the 62 control group participants (98.4%) logged-on to the Thrive alcohol intervention. 

### 3.5. Program Acceptability 

Of the 62 participants in the intervention group, 52 completed the process evaluation survey (83.9% response rate). Results for the acceptability measures include the 49 of these 52 participants who logged-on to the EATS website. All responses are included for the questions which asked participants their reasons for not completing intervention components. The mean satisfaction with EATS was 4.0 ± 0.7, and the mean score for EATS meeting expectations was 4.0 ± 0.8 (Max 5 = Very satisfied) (Table 2). The majority of participants (75.5%) indicated that the time taken to complete EATS was ‘a good amount of time’. The percentage of participants who saved a copy of the relevant output for each of the components included 24.5% for the quiz feedback, 24.5% saved a copy of their goals, and 18.4% saved a copy of their strategies. 

The reasons participants reported for not completing the quiz were lack of time and not seeing it. The main reasons participants reported for not completing the goal setting and creating strategies components were lack of time, feeling like they didn’t need to set goals or strategies (e.g., *“Didn’t see a need”*) or they could set goals/strategies without using the website (e.g., *“I feel I have the ability to create my own strategies”*), and not being interested in these components (e.g., *“Wasn’t interested”*, *“Too much effort”)*. 

### 3.6. Measures of Potential Contamination

Seventy-percent of intervention participants and 46% of control participants reported that they had made changes to their eating behavior in the three months since baseline. Of those in the intervention group, 33% reported that they had used only the EATS intervention to help them change behavior, 21% reported using EATS and other sources of information e.g., websites or smartphone apps, 30% reported using other sources of information e.g., websites or smartphone apps only, and 15% reported using no strategies to help change their eating behaviors. 

### 3.7. Estimation of Treatment Effect

Results of the ITT analysis exploring the difference between groups from baseline to three months are presented in Table 3. No significant differences were observed between or within groups for the outcome of diet quality (total ARFS). A significant difference in favor of the intervention group was observed for the percentage energy from discretionary foods (−4.8%, 95%CI −8.6, −1.1, *p* = 0.012, Cohen’s *d* = −0.34). Significant within group changes were observed for the control group for increased frequency of breakfast consumption, increased self-efficacy for consuming fruit and decreased frequency of alcohol consumption, however between group differences were not significant for these outcomes. No other significant differences were observed between or within groups for self-efficacy, quality of life or well-being outcomes. Results of the completers analysis is presented in Supplementary Table S1 and they were consistent with ITT. Results of the sensitivity analysis with plausible energy reporters only (*n* = 78) is presented in Supplementary Table S2 and they were also consistent with ITT. 

## 4. Discussion 

This pilot RCT evaluated the feasibility and preliminary efficacy of the EATS brief web-based nutrition intervention for young adult university students. Recruitment methods were successful and a high retention rate achieved, suggesting that recruiting via a range of methods and using existing university communication infrastructure, and using online surveys and minimal reimbursement for data collection, are feasible methods. Intervention usage and acceptability (satisfaction, usability, and appropriateness) were also high, with most participants completing the intervention components and high average ratings across acceptability measures. In terms of the impact evaluation, there were no significant between group differences for diet quality over time, however there was a significant difference in favor of the intervention group for discretionary foods intake (i.e., a decrease in percentage energy per day from discretionary foods). Overall, the positive findings provide support for further development and evaluation of the EATS intervention. 

The current study successfully recruited 124 young adult university students over a five-week period. Females and students studying health and medicine degrees were over-represented within the sample despite attempts to recruit a diverse sample and maximize male participants. However, the sample was representative of the UON student body in terms of similar proportions of domestic and Indigenous students [36,37]. The greater interest among female students could potentially be explained by gender differences in the perceived importance of, and motivation for, healthy eating, as males generally have been found to place less importance on healthy eating [2]. Although the recruitment period was extended and the target of 30% male participants was not reached, the recruitment was still considered successful overall based on the total number of young adults recruited in a relatively short duration. In total, 303 individuals responded to the recruitment materials over the five weeks, and 250 were eligible to participate, equaling twice the target sample size. As the resources available limited the number of participants able to be enrolled, a participation rate cannot be calculated, however this high response to recruitment is encouraging. In comparison, a review of the strategies used to recruit young adults in to weight gain prevention programs by Lam et al. found that for studies with sample sizes greater than 100, recruitment took between nine months and three years [34]. Of the 26 studies in the review, 23 also recruited from universities or colleges, including 21 exclusively [34]. A range of recruitment strategies were effective in recruiting the current study sample, including advertising via university teaching staff, who promoted the study in class and through the online learning management system, and via university social media pages and posters displayed across campuses. The number of individuals interested peaked in line with when university staff were asked to advertise the study, which was a secondary recruitment method. The difference in response via these methods could be explained by the fact that utilizing teaching staff and infrastructure to communicate with students had a much wider reach than social media, with only a small proportion of the total student body following the UON social media accounts. The review by Lam et al., as well as a scoping review of social media use in recruiting for medical research studies, also found limited effectiveness in recruiting via social media [34,38]. Instead, Lam et al. recommended using a range of active and passive strategies to recruit young adults [34]. Overall, the evidence supports that utilizing the university setting is an effective way to reach young adults. In terms of recruitment methods, using a range of methods may be most effective, including utilizing existing infrastructure and methods for communicating with students (e.g., online learning platforms). 

The retention rate achieved in the current study was good, with 73% providing complete data at 3-month follow up, or 86% including those who provided partial data at three months. Comparatively, the average retention rate across 41 studies in a systematic review of nutrition, physical activity and weight management interventions among university students was 77%, with most studies involving pre and post intervention assessments [11]. The high retention rate in the current study was achieved with minimal reimbursements, including AUD $10 gift vouchers for completing measures at baseline and follow-up, and with few reminders needed for the majority of participants; almost half completed follow up surveys without a reminder and within one week (i.e., before the first scheduled reminder). For the remainder, between 1–7 reminders across different communication methods (email, text message and phone calls) were used. For the process evaluation survey, more reminders were used to achieve the 84% completion, as well as extending the time period for survey completion and including the process evaluation survey within the follow up survey for non-completers. However, intervention participants did not receive anything additional for completing the process evaluation survey. Additionally, the timing of the process evaluation and follow up measures may have limited their completion. For some participants, process evaluation and follow up measures coincided with the break between semesters and the end of semester exam period. Previous studies among young adults and university students have found that incentives were needed to achieve adequate retention and completion of data collection. Monetary incentives and, for university students, receiving course credit have both been found to be effective [12,34,39]. In terms of monetary incentives, the value of these in previous studies varies greatly as it is usually dependent on available funding, however the value is often higher than the reimbursements in the current study [34,39]. For example, in the CHOICES weight gain prevention trial for young adult college students, $US100 gift vouchers were provided as incentives for completing baseline measures and at three follow up time points up to 24 months, where retention rate at 24 months was 84% [39]. While larger incentives may be more effective or necessary for longer-term follow-up, low or no cost incentives are preferable and more feasible. Low or no cost incentives, such as minimal monetary incentives and course credit, and the use of reminders across different communication methods, may be effective retention strategies for young adult university students. Limiting the data collection to include only essential measures, and where appropriate using online and brief questionnaires or tools may also enhance retention by limiting participant burden [39]. 

Overall, participants were highly engaged with the EATS intervention, in terms of usage and high acceptability ratings. Although engagement in eHealth is ambiguous to quantify [40], engagement in the current study was considered high as most of the participants completed the key intervention components (quiz, goal setting and creating strategies) and rated them consistently high (range 3.9–4.3) for their usefulness, relevance and motivation to positively change behavior. Most participants used EATS in a single session as was the intention, and most also indicated that it took a good amount of time to complete. The quiz component with personalized feedback had the highest usage and was rated most highly in terms of relevance and motivation to increase consumption of healthy foods. Providing feedback on behavior has been demonstrated to be an effective behavior change technique in many previous interventions [11,17], and this finding is consistent with that. Around half of the participants used the goal setting and creating strategies components. Among non-users, the main reasons participants reported for not using these components were lack of time, that they didn’t feel they needed to set goals or strategies or that they thought they could do this without the help of the website. In regards to lack of time, those who used EATS over more than one session were not more likely to complete all components. It could be that other factors, such as motivation or interest to change eating behaviors, were the drivers here of a perceived lack of time. Using the quiz component only may have been enough for some participants, meaning they optimally engaged but they could also be considered as having lower total engagement [41]. Further, if participants set goals or strategies without the use of the website this is not captured, but could still be considered a positive effect of the intervention. Overall, there was high satisfaction with, and acceptability of, this brief web-based intervention approach. 

The preliminary efficacy results are promising, despite the study not being powered to detect between-group differences in outcomes. Changes in diet quality, and intakes of fruit, vegetables and breakfast were non-significant, however moved in the desired direction. A significant between group difference was observed for the percentage energy/day from discretionary foods, with intervention participants reducing their intake by 4.8% of energy/day compared with control participants (*d =* 0.34). In comparison, a systematic review of brief nutrition interventions in adults found that among studies that were effective in reducing fat intake, the percentage energy/day from total fats decreased by between 1 and 8% in brief intervention groups compared with controls [17]. Comparatively, in a non-brief intervention among young adult male university students, intervention participants reduced their percentage energy/day from fats by 3.2% compared with controls, following 4-weeks of nutrition counseling [42]. In the Connecting Health and Technology (CHAT) study among young adults, two non-brief interventions aiming to reduce discretionary foods consumption and increase fruit and vegetable consumption (feedback and weekly text messages or feedback only) were compared with a no intervention control group in a six month RCT [43]. Participants in the feedback and text message and feedback only groups decreased discretionary foods intake by 0.3 and 0.4 serves per day respectively, however the difference was non-significant [43]. The current study results are promising in comparison with other studies among university students and young adults due to the benefits of a brief, web-based intervention approach. A brief intervention has a limited time commitment on the part of participants and administrators, especially when delivered via eHealth, making it more acceptable and accessible. There is also the potential benefit to cost-effectiveness, in that the mode of delivery (automated website) minimizes the ongoing resources and therefore cost required. 

The strengths of this study include the RCT design, including stratification by gender to mitigate the potential bias of a predominantly female sample, the mix of objective measures and validated tools used in measuring feasibility and preliminary efficacy, and the high retention rate achieved. Further, a strong methodological approach was undertaken for intervention development [20]. In terms of limitations, this was a pilot RCT and therefore preliminary efficacy results are indicative rather than conclusive. The short term follow up also limits the interpretation and generalizability of the findings, and future evaluation of the EATS intervention should include a long term follow up. Additionally, due to multiple data sources being used for the collection of intervention usage data, not all data could be individually matched to participants, and this limited the ability to compare the objective with self-reported data for intervention usage and program acceptability for consistency. 

## 5. Conclusions

Young adults are typically a challenging group to reach and engage in health behavior interventions. The EATS intervention utilized the university setting and a web-based approach to enhance reach, a brief intervention approach to address the barrier of lack of time, and a participatory research model to improve reach and engagement. The results of this pilot RCT demonstrate that there is potential for a brief web-based nutrition intervention for young adult university students in terms of acceptability and preliminary efficacy. 

## Figures and Tables

**Table:** 

Recruitment strategy	Week 1	Week 2	Week 3	Week 4	Week 5	Week 6
University of Newcastle Social Media	✓	✓	✓			
Posters on campus	✓	✓	✓	✓	✓	✓
Digital signage on campus		✓	✓			
University of Newcastle Health Promotion Working Group	✓				✓	
EATS Steering committee	✓				✓	
University of Newcastle Teaching staff				✓		

**Table 1:** Baseline characteristics of young adult university students participating in the EATS pilot randomized controlled trial (*n* = 124).

Characteristic	Total (*n* = 124)	Intervention (*n* = 62)	Control (*n* = 62)
	Mean ± SD or %(*n*)		
**Age (years) (mean ± SD)**	22.4 ± 4.0	21.8 ± 3.2	23.0 ± 4.6
**Gender %(*n*)**			
Female	72.6 (90)	71.0 (44)	74.2 (46)
Male	27.4 (34)	29.0 (18)	25.8 (16)
**Australian born %(*n*)**	83.1 (103)	77.4 (48)	88.7 (55)
**Aboriginal or Torres Strait Islander %(*n*)**	3.2 (4)	1.6 (1)	4.8 (3)
**IRSAD (mean ± SD) ^a^**	6.0 ± 2.5	6.0 ± 2.4	6.0 ± 2.6
**Marital status %(*n*)**			
Never married	85.5 (106)	88.7 (55)	82.3 (51)
Married	8.9 (11)	4.8 (3)	12.9 (8)
Defacto	4.8 (6)	6.5 (4)	3.2 (2)
Separated/divorced/widowed	0.8 (1)	0 (0)	1.6 (1)
**Living situation %(*n*)**			
Own home	5.7 (7)	4.8 (3)	6.5 (4)
Parents home	32.3 (40)	33.9 (21)	30.7 (19)
On-campus	16.1 (20)	19.4 (12)	12.9 (8)
Renting	41.9 (52)	38.7 (24)	45.2 (28)
Boarding/homestay	2.4 (3)	1.6 (1)	3.2 (2)
Irregular	1.6 (2)	1.6 (1)	1.6 (1)
**Paid work (hours/week) (mean ± SD)**	8.4 ± 10.1	7.6 ± 10.0	9.3 ± 10.2
**Receiving financial support %(*n*)**	76.6 (95)	72.6 (45)	80.7 (50)
**Student type %(*n*)**			
Undergraduate	87.1 (108)	90.3 (56)	83.9 (52)
Postgraduate (course)	0.8 (1)	0 (0)	1.6 (1)
Postgraduate (HDR)	7.3 (9)	4.8 (3)	9.7 (6)
Enabling	4.0 (5)	3.2 (2)	4.8 (3)
Non-award	0.8 (1)	1.6 (1)	0 (0)
**Domestic/International %(*n*)**			
Domestic	90.3 (112)	87.1 (54)	93.6 (58)
International	9.7 (12)	12.9 (7)	6.4 (4)
**Number of years studying %(*n*)**			
1st year	32.3 (40)	29.0 (18)	35.5 (22)
2nd year	21.8 (27)	25.8 (16)	17.7 (11)
3rd year	18.6 (23)	21.0 (13)	16.1 (10)
4th year	16.9 (21)	12.9 (8)	21.0 (13)
5th year or later	10.5 (13)	11.3 (7)	9.7 (6)
**Faculty of study %(*n*)**			
Business & law	7.3 (9)	8.1 (5)	6.5 (4)
Education & arts	21.0 (26)	9.7 (6)	32.3 (20)
Engineering/built environment	7.3 (9)	4.8 (3)	9.7 (6)
Health & medicine	46.8 (58)	50.0 (31)	43.6 (27)
Science	15.3 (19)	24.2 (15)	6.5 (4)
English language/foundation studies	2.4 (3)	3.2 (2)	1.6 (1)
**Dietary intake**			
ARFS (/73) (mean ± SD)	30.5 ± 9.8	32.2 ± 10.1	28.9 ± 9.3
Fruit ARFS sub-scale (/12) (mean ± SD)	4.9 ± 2.8	5.3 ± 2.9	4.4 ± 2.7
Fruit (grams/day)	232.2 ± 186.3	243.5 ± 219.6	220.9 ± 146.5
Fruit (% energy/day)	7.6 ± 6.0	7.6 ± 5.6	7.7 ± 6.4
Vegetables ARFS sub-scale (/21) (mean ± SD)	10.9 ± 4.7	11.4 ± 4.9	10.5 ± 4.4
Vegetables (grams/day)	265.9 ± 160.7	283.4 ± 163.2	248.5 ± 157.6
Vegetables (% energy/day)	8.0 ± 5.1	8.3 ± 5.0	7.6 ± 5.2
Discretionary foods (% energy/day)	36.6 ± 14.0	36.8 ± 13.4	36.3 ± 14.8
Breakfast %(*n*)			
Never	4.0 (5)	3.2 (2)	4.8 (3)
1–2 days/week	10.5 (13)	9.7 (6)	11.3 (7)
3–4 days/week	18.6 (23)	16.1 (10)	21.0 (13)
5 or more days/week	66.9 (83)	71.0 (44)	62.9 (39)
**Diet self-efficacy scores (mean ± SD)**			
Fruit	3.2 ± 0.9	3.1 ± 1.0	3.3 ± 0.8
Vegetables	2.8 ± 0.9	2.8 ± 1.0	2.7 ± 0.9
Takeaway foods	3.1 ± 0.9	3.1 ± 1.0	3.0 ± 0.8
EDNP snack foods	2.6 ± 1.0	2.8 ± 1.0	2.5 ± 1.0
Sugar sweetened drinks	3.4 ± 0.9	3.4 ± 1.0	3.4 ± 0.8
Alcohol	3.4 ± 1.0	3.4 ± 1.0	3.4 ± 1.0
Breakfast	3.4 ± 1.0	3.3 ± 1.1	3.4 ± 0.9
**Alcohol intake %(*n*)**			
Exceeding single occasion risk	32.3 (40)	33.9 (21)	30.7 (19)
Exceeding lifetime risk	50.8 (63)	51.6 (32)	50.0 (31)
**BMI (mean ± SD)**	24.6 ± 4.8	24.9 ± 5.3	24.2 ± 4.3
Underweight %(*n*)	3.2 (4)	3.2 (2)	3.2 (2)
Healthy weight %(*n*)	57.3 (71)	53.2 (33)	61.3 (38)
Overweight %(n)	26.6 (33)	29.0 (18)	24.2 (15)
Obese %(*n*)	12.9 (16)	14.5 (9)	11.3 (7)
**WHO-5 score (mean ± SD) ^b^**	14.7 ± 4.3	15.2 ± 4.0	14.3 ± 4.6
**Q-LES-Q-SF score (mean ± SD) ^c^**	51.3 ± 7.5	52.6 ± 7.1	50.0 ± 7.7

IRSAD, Index of Relative Socioeconomic Advantage and Disadvantage; ARFS, Australian Recommended Food Score; EDNP, Energy-Dense Nutrient Poor; Q-LES-Q-SF, Quality of Life Enjoyment and Satisfaction Questionnaire. ^a^ IRSAD only available for participants living in Australia prior to university enrolment (*N* = 112); ^b^ WHO-5 score range = 0–25, higher score indicating greater well-being; ^c^ Q-LES-Q-SF score range 0–70, higher score indicating greater quality of life.

**Table 2:** Acceptability of the Eating Advice To Students (EATS) brief web-based nutrition intervention components (*N* = 49).

**Acceptability Measures**	**Website Overall**		
Useful information about healthy eating	4.1 ± 0.7		
Relevant information about healthy eating	4.2 ± 0.6		
New information about healthy eating	3.5 ± 1.0		
Motivated me to eat more healthy foods	3.8 ± 0.8		
Motivated me to eat less discretionary foods	3.8 ± 0.7		
Was easy to use	4.3 ± 0.7		
Was visually appealing	4.3 ± 0.6		
**Acceptability Measures**	**Quiz Component**	**Goal Setting Component**	**Creating Strategies Component**
Was useful	4.1 ± 0.6	3.9 ± 0.8	4.1 ± 0.7
Was relevant	4.2 ± 0.7	4.1 ± 0.7	4.1 ± 0.7
Was easy to use	4.4 ± 0.6	4.3 ± 0.8	4.3 ± 0.7
Motivated me to eat more healthy foods	4.1 ± 0.8	3.9 ± 0.8	3.9 ± 0.9
Motivated me to eat less discretionary foods	4.0 ± 0.7	4.0 ± 0.8	3.9 ± 0.8
Made me more aware of what I was eating	4.2 ± 0.8	NA	NA
Provided me with useful examples for setting goals/creating strategies	NA	4.2 ± 0.8	4.1 ± 0.8

All measures scored from 0–5, NA indicates Not Applicable (i.e., was not assessed) for this program component. Numbers providing data for each component include website overall *N* = 49, quiz component *N* = 47, goal setting component N = 40, creating strategies component *N* = 38.

**Table 3:** Primary and secondary outcome changes in young adult university students (n = 124) participating in the Eating Advice To Students (EATS) brief web-based nutrition intervention pilot RCT from baseline to three months using intention-to-treat (ITT) analysis.

Outcome	Mean Change from Baseline to 3-Months (95%CI)				Mean Difference between Groups (95% CI)	Group by Time *p*-Value	Effect Size (Cohen’s *d*)
	Intervention (*n* = 62)	*p*-Value	Control (*n* = 62)	*p*-Value			
Diet quality (ARFS)	0.1 (−1.9, 2.1)	0.927	1.1 (−0.8, 3.0)	0.243	−1.0 (−3.8, 1.7)	0.468	-0.10
Fruit (ARFS sub-scale score)	0.2 (−0.5, 0.8)	0.620	−0.1 (−0.7, 0.6)	0.834	0.2 (−0.7, 1.1)	0.613	0.08
Fruit (grams/day)	0.02 (−58.5, 58.5)	0.999	9.5 (−19.8, 38.7)	0.525	−9.5 (−74.9, 55.9)	0.776	-0.05
Fruit (grams/day) (adjusted model) ^a^	21.7 (−13.6, 56.9)	0.228	7.6 (−24.6, 39.8)	0.645	14.1 (−33.7, 61.9)	0.563	0.09
% energy from fruit	0.4 (−1.1, 2.0)	0.606	0.1 (−1.3, 1.6)	0.870	0.3 (−1.8, 2.4)	0.790	0.05
Vegetable (ARFS sub-scale score)	0.1 (−1.0, 1.2)	0.870	0.7 (−0.3, 1.8)	0.165	−0.6 (−2.2, 0.9)	0.410	−0.14
Vegetable (grams/day)	18.8 (−19.1, 56.6)	0.332	−8.1 (−43.3, 27.0)	0.650	26.9 (−24.7, 78.5)	0.307	0.17
% energy from vegetables	0.3 (−0.8, 1.5)	0.561	0.3 (−0.8, 1.3)	0.612	0.1 (−1.5, 1.6)	0.935	0.01
% energy from discretionary foods	−3.6 (−6.4, −0.8)	**0.010**	1.2 (−1.3, 3.8)	0.349	−4.8 (−8.6, −1.1)	**0.012**	−0.34
QLESQ total score	0.6 (−1.3, 2.5)	0.561	−0.04 (−1.9, 1.8)	0.962	0.6 (−2.3, 3.2)	0.652	0.08
WHO-5 score	0.3 (−0.8, 1.3)	0.580	−0.4 (−1.4, 0.6)	0.422	0.7 (−0.7, 2.2)	0.339	0.17
	**Below categorical variables presented as odds ratio ^b^**						
Breakfast (frequency consumed)	1.7 (0.4, 6.8)	0.475	7.4 (1.7, 32.1)	**0.008**	0.2 (0.03, 1.7)	0.153	−0.35
Alcohol (quantity consumed)	1.5 (0.6, 3.8)	0.383	2.5 (1.0, 6.3)	**0.044**	0.6 (0.2, 2.1)	0.424	−0.42
Fruit self-efficacy (confidence score)	0.8 (0.3, 1.7)	0.516	0.3 (0.1, 0.6)	**0.002**	3.0 (0.9, 10.0)	0.071	0.26
Vegetables self-efficacy (confidence score)	1.0 (0.4, 2.1)	0.927	0.8 (0.4, 1.6)	0.455	1.3 (0.4, 3.8)	0.657	0.06
Takeaway foods self-efficacy (confidence score)	0.6 (0.3, 1.4)	0.240	0.6 (0.3, 1.3)	0.188	1.0 (0.3, 1.0)	0.977	0.00
EDNP snack foods self-efficacy (confidence score)	0.7 (0.3, 1.6)	0.393	1.3 (0.6, 2.8)	0.462	0.5 (0.2, 1.6)	0.261	−0.15
Sugar sweetened drinks self-efficacy (confidence score) ^c^	0.8 (0.3, 2.0)	0.619	0.8 (0.3, 1.9)	0.614	1.0 (0.3, 3.5)	0.966	−0.01
Alcohol self-efficacy (confidence score)	0.2 (0.05, 0.5)	**0.002**	0.9 (0.3, 2.5)	0.800	0.2 (0.04, 0.8)	**0.031**	−0.42
Breakfast self-efficacy (confidence score)	0.5 (0.2, 1.5)	0.245	0.9 (0.4, 2.5)	0.902	0.6 (0.1, 2.4)	0.447	−0.13

ARFS, Australian Recommended Food Score; EDNP, Energy-Dense Nutrient Poor; QLESQ, Quality of Life, Enjoyment & Satisfaction Questionnaire; WHO-5, World Health Organization-Five Well-being Index. ^a^ Adjusted model with *n* = 1 intervention participant removed from analysis due to outlier (1278 grams/day at baseline and 218 grams/day at follow-up). ^b^ Categorical variables are presented as OR of moving up a category (i.e., desirable direction), with the exception of alcohol intake which is odds of moving down a category. ^c^ Proportional odds assumption not met for this model, however alternative modeling did not change the result or interpretation. Significant *p*-values are indicated in bold.

## Supplementary Materials

The following are available online at https://www.mdpi.com/2072-6643/11/4/905/s1, Supplementary Table S1: Primary and secondary outcome changes in young adult university students (*n* = 106) who completed the Eating Advice To Students (EATS) brief online nutrition intervention pilot RCT from baseline to three months; Supplementary Table S2: Primary and secondary outcome changes in young adult university students (*n* = 78) who reported plausible energy intake at baseline in the Eating Advice To Students (EATS) brief web-based nutrition intervention pilot RCT from baseline to three months.
