# Supplementary material for: Feasibility and Preliminary Efficacy of the Eating Advice to Students (EATS) Brief Web-Based Nutrition Intervention for Young Adult University Students: A Pilot Randomized Controlled Trial
Source: Nutrients. 2019 Apr 23;11(4):905. doi: 10.3390/nu11040905 (PMC6520699; doi:10.3390/nu11040905)
Supplement: Supplementary file 1 [file nutrients-11-00905-s001.pdf]

**Table S1** Primary and secondary outcome changes in young adult university students (n=106) who completed the Eating Advice To Students (EATS) brief web-based nutrition intervention pilot RCT from baseline to 3 months

| **Outcome** | **Mean change from baseline to 3-months (95%CI)** | | | | **Mean difference between groups (95% CI)** | **Group*Time p-value** | **Effect size** |
|---|---|---|---|---|---|---|---|
| | **Intervention (n=42 or 50)** | **p-value** | **Control (n=50 or 54)** | **p-value** | | | |
| Diet quality (ARFS) [a] | -0.3 (-2.4, 1.7) | 0.750 | 1.1 (-0.8, 2.9) | 0.269 | -1.4 (-4.2, 1.4) | 0.326 | -0.16 |
| Fruit (ARFS sub-scale score) [a] | 0.1 (-0.6, 0.8) | 0.731 | -0.1 (-0.7, 0.5) | 0.705 | 0.2 (-0.7, 1.2) | 0.611 | 0.09 |
| Fruit (grams/day) [a] | -4.8 (-53.3, 43.7) | 0.846 | 4.7 (-39.8, 49.1) | 0.837 | -9.4 (-75.2, 56.3) | 0.778 | -0.05 |
| Fruit (grams/day) (adjusted model) [a, b] | 20.9 (-14.6, 56.4) | 0.248 | 4.7 (-27.5, 36.8) | 0.777 | 16.3 (-31.6, 64.2) | 0.505 | 0.11 |
| % energy from fruit | 0.2 (-1.4, 1.8) | 0.812 | 0.2 (-1.3, 1.6) | 0.827 | 0.03 (-2.1, 2.2) | 0.978 | 0.01 |
| Vegetable (ARFS sub-scale score) [a] | -0.2 (-1.3, 1.0) | 0.744 | 0.8 (-0.2, 1.8) | 0.135 | -1.0 (-2.5, 0.6) | 0.211 | -0.22 |
| Vegetable (grams/day) [a] | 17.4 (-21.3, 56.1) | 0.378 | -10.7 (-46.1, 24.8) | 0.556 | 28.1 (-24.4, 80.6) | 0.294 | 0.18 |
| % energy from vegetables [a] | 0.3 (-0.9, 1.4) | 0.650 | 0.4 (-0.7, 1.4) | 0.497 | -0.1 (-1.6, 1.4) | 0.900 | -0.02 |
| % energy from discretionary foods [a] | -3.0 (-5.8 -0.2) | **0.033** | 1.6 (-0.9, 4.2) | 0.212 | -4.6 (-8.4, -0.9) | **0.016** | -0.37 |
| QLESQ total score [a] | 0.2 (-1.8, 2.1) | 0.870 | -0.2 (-2.0, 1.7) | 0.859 | 0.3 (-2.3, 3.0) | 0.810 | 0.05 |
| WHO-5 score [a] | 0.1 (-0.9, 1.2) | 0.797 | -0.6 (-1.6, 0.5) | 0.272 | 0.7 (-0.8, 2.2) | 0.343 | 0.17 |
| ***Below categorical variables presented as odds ratio*** [c] | | | | | | | |
| Breakfast (frequency consumed) [a] | 1.3 (0.3, 5.8) | 0.734 | 7.1 (1.6, 31.4) | **0.010** | 0.2 (0.02, 1.5) | 0.109 | -0.41 |
| Alcohol (quantity consumed) [d] | 1.5 (0.6, 3.7) | 0.430 | 2.7 (1.1, 6.6) | **0.035** | 0.5 (0.2, 2.0) | 0.357 | 0.30 |

| | | | | | | | |
|---|---|---|---|---|---|---|---|
| Fruit self-efficacy (confidence score) [d] | 0.8 (0.3, 1.9) | 0.605 | 0.3 (0.1, 0.6) | **0.002** | 3.1 (0.9, 10.6) | 0.065 | 0.27 |
| Vegetables self-efficacy (confidence score) [d] | 1.1 (0.5, 2.4) | 0.846 | 0.7 (0.3, 1.6) | 0.435 | 1.5 (0.5, 4.4) | 0.501 | 0.09 |
| Takeaway foods self-efficacy (confidence score) [d] | 0.6 (0.2, 1.3) | 0.191 | 0.6 (0.3, 1.2) | 0.163 | 1.0 (0.3, 3.0) | 0.974 | 0.00 |
| EDNP snack foods self-efficacy (confidence score) [d] | 0.7 (0.3, 1.5) | 0.341 | 1.3 (0.6, 2.7) | 0.541 | 0.5 (0.2, 1.6) | 0.267 | -0.15 |
| Sugar sweetened drinks self-efficacy (confidence score) [d, e] | 0.5 (0.2, 1.5) | 0.233 | 0.7 (0.3, 1.7) | 0.449 | 0.7 (0.2, 2.8) | 0.670 | -0.07 |
| Alcohol self-efficacy (confidence score) [d] | 0.1 (0.04, 0.5) | **0.002** | 0.9 (0.3, 2.6) | 0.855 | 0.2 (0.03, 0.8) | **0.023** | -0.45 |
| Breakfast self-efficacy (confidence score) [d] | 0.5 (0.2, 1.4) | 0.187 | 1.0 (0.4, 2.5) | 0.937 | 0.5 (0.1, 2.1) | 0.364 | -0.16 |

ARFS, Australian Recommended Food Score; EDNP, Energy-Dense Nutrient Poor; QLESQ, Quality of Life, Enjoyment & Satisfaction Questionnaire; WHO-5, World Health Organization-Five Well-being Index. [a] n=42 for intervention group and n=50 for control group (i.e. those who completed the Australian Eating Survey Food Frequency Questionnaire at follow up) [b] Adjusted model with n=1 intervention participant removed from analysis due to outlier (1278 grams/day at baseline and 218 grams/day at follow-up). [c] Categorical variables are presented as OR of moving up a category (i.e. desirable direction), with the exception of alcohol intake which is odds of moving down a category. [d] n=50 for intervention group and n=54 for control group (i.e. those who completed the other survey questions at follow up) [e] Proportional odds assumption not met for this model, however alternative modelling did not change the result or interpretation

**Table S2** Primary and secondary outcome changes in young adult university students (n=78) who reported plausible energy intake at baseline in the Eating Advice To Students (EATS) brief web-based nutrition intervention pilot RCT from baseline to 3 months

| Outcome | Mean change from baseline to 3-months (95%CI) | | | | Mean difference between groups (95% CI) | Group*Time p-value | Effect size (Cohen's *d*) |
|---|---|---|---|---|---|---|---|
| | Intervention (n=39) | p-value | Control (n=39) | p-value | | | |
| Diet quality (ARFS) | 0.6 (-1.7, 3.0) | 0.587 | 0.2 (-1.9, 2.3) | 0.843 | 0.4 (-2.7, 3.6) | 0.791 | 0.05 |
| Fruit (ARFS sub-scale score) | 0.4 (-0.4, 1.1) | 0.373 | -0.1 (-0.8, 0.6) | 0.765 | 0.5 (-0.6, 1.5) | 0.392 | 0.18 |
| Fruit (grams/day) | 12.7 (-33.4, 58.8) | 0.589 | 10.0 (-32.5, 52.5) | 0.646 | 2.8 (-60.0, 65.5) | 0.931 | 0.02 |
| % energy from fruit | 0.9 (-0.7, 2.5) | 0.289 | 1.1 (-0.4, 2.7) | 0.139 | -0.3 (-2.5, 2.0) | 0.818 | -0.06 |

| | | | | | | | |
|---|---|---|---|---|---|---|---|
| Vegetable (ARFS sub-scale score) | 0.2 (-1.1, 1.6) | 0.745 | 0.3 (-1.0, 1.5) | 0.662 | -0.1 (-1.9, 1.8) | 0.954 | -0.01 |
| Vegetable (grams/day) | 14.2 (-33.3, 61.9) | 0.556 | -30.0 (-74.1, 14.1) | 0.182 | 44.3 (-20.5, 109.2) | 0.180 | 0.28 |
| % energy from vegetables | 0.5 (-0.7, 1.8) | 0.399 | -0.1 (-1.2, 1.1) | 0.915 | 0.6 (-1.1, 2.3) | 0.489 | 0.13 |
| % energy from discretionary foods | -4.1 (-7.8, -0.5) | **0.027** | 1.6 (-1.8, 5.0) | 0.347 | -5.7 (-10.7, -0.8) | **0.024** | -0.45 |
| ***Below categorical variables presented as odds ratio*** [b] | | | | | | | |
| Breakfast (frequency consumed) | 1.1 (0.1, 10.0) | 0.914 | 4.5 (0.8, 25.6) | 0.091 | 0.3 (0.02, 4.2) | 0.334 | -0.33 |

ARFS, Australian Recommended Food Score. Plausible energy reporters were identified from baseline energy intake using the Goldberg cutoffs [1], with basal metabolic rate calculated using the Schofield equation [2] and a physical activity level of 1.55 used for all participants. Participants with an energy intake: basal metabolic rate <0.97 were classified as under-reporters and those with energy intake: basal metabolic rate >2.48 as over-reporters. There was no significant difference in the number of plausible reporters between groups (intervention n=39, control n=39).

## References


1. Black, A.E. Critical evaluation of energy intake using the goldberg cut-off for energy intake:Basal metabolic rate. A practical guide to its calculation, use and limitations. *Int J Obes Relat Metab Disord* **2000**, *24*, 1119-1130.
2. Schofield, W.N. Predicting basal metabolic rate, new standards and review of previous work. *Hum Nutr Clin Nutr* **1985**, *39 Suppl 1*, 5-41.
